# Stathmin 1 across neuroendocrine neoplasms: biological, pathological and translational insights

**DOI:** 10.3389/fendo.2026.1846997

**Published:** 2026-08-06

**Authors:** Anna La Salvia, Alessandro Tanca, Germano Gaudenzi, Giulia Pecora, Antongiulio Faggiano, Giovanni Vitale, Paolo Cossu-Rocca, Giuseppe Fanciulli

**Affiliations:** 1 National Center for Drug Research and Evaluation, National Institute of Health (ISS), Rome, Italy; 2 Department of Biomedical Sciences, University of Sassari, Sassari, Italy; 3 Unit of Microbiology and Virology, University Hospital of Sassari, Sassari, Italy; 4 Laboratory of Geriatric and Oncologic Neuroendocrinology Research, IRCCS, Istituto Auxologico Italiano, Cusano Milanino, Milan, Italy; 5 Endocrinology Unit, Department of Clinical and Molecular Medicine, The European Neuroendocrine Tumor Society (ENETS) Center of Excellence, Sant’Andrea Hospital, Sapienza University of Rome, Rome, Italy; 6 Department of Medical Biotechnology and Translational Medicine, University of Milan, Milan, Italy; 7 Department of Medicine, Surgery and Pharmacy, University of Sassari, Sassari, Italy; 8 Surgical Pathology Unit, “Giovanni Paolo II” Hospital, Local Health Authority (ASL) Gallura, Olbia, Italy; 9 Department of Medicine, Surgery and Pharmacy, University of Sassari - University Hospital (AOU) of Sassari, Sassari, Italy

**Keywords:** biomarker, neuroendocrine carcinoma, neuroendocrine neoplasms, neuroendocrine tumours, pathology, stathmin 1 (STMN1), translational oncology, tumour aggressiveness

## Abstract

Stathmin 1 (STMN1) is a cytosolic phosphoprotein involved in microtubule dynamics, mitotic progression and cytoskeleton-dependent cellular behaviour. In multiple human malignancies, dysregulated STMN1 expression has been associated with aggressive clinicopathological features, adverse outcomes and, in selected settings, potential therapeutic vulnerability. Its significance in neuroendocrine neoplasms (NENs), however, has not been critically synthesised across anatomical sites. This narrative review examined the available literature on STMN1 in NENs across pulmonary, gastroenteropancreatic, adrenal, cutaneous, pituitary and thyroid settings, with the specific aim of mapping site-specific expression patterns and appraising whether reported associations with aggressive clinicopathological features were descriptive, independently validated, or functionally supported. A literature search was conducted in PubMed, Web of Science, and Scopus from inception to 25 January 2026. Twelve original studies met the inclusion criteria and were narratively synthesised. The available evidence indicates that STMN1 is not a uniform marker of neuroendocrine differentiation across tumour types, but rather a context-dependent signal more often associated with proliferative activity, high-grade morphology and malignant behaviour. The strongest evidence currently derives from pulmonary neuroendocrine neoplasms, where STMN1 has shown diagnostic utility in tissue and bronchoalveolar lavage and where preclinical targeting data are available. Outside the lung, however, the literature is limited and methodologically heterogeneous, and the evidentiary weight varies from descriptive expression studies to functional preclinical observations. Overall, STMN1 emerges as a recurrent molecular signal in several NEN settings, particularly in aggressive and high-grade disease contexts. Its causal biological relevance and clinical value, including potential implications for treatment sensitivity and tumour dissemination, remain to be demonstrated in studies specifically designed for prognostic and therapeutic validation. Current therapeutic evidence remains preclinical and should be regarded as hypothesis-generating rather than clinically actionable.

## Graphical Abstract

**Figure:**
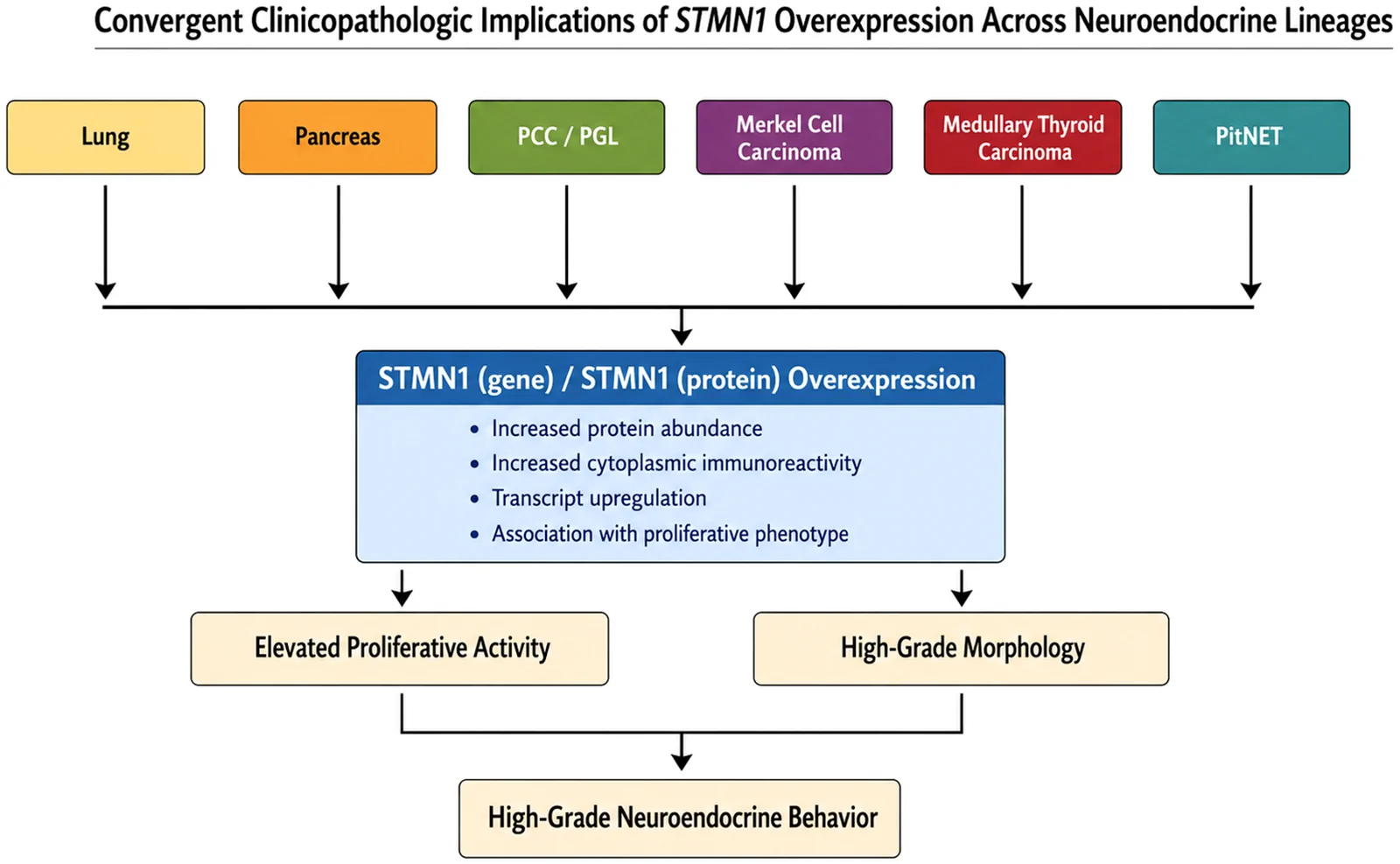
PCC, pheochromocytoma; PGL, paraganglioma, PitNET, pituitary neuroendocrine tumour.

## 1 Introduction

Neuroendocrine neoplasms (NENs) comprise a heterogeneous group of epithelial neoplasms arising in multiple anatomical sites, including the gastroenteropancreatic (GEP) tract, lung, adrenal medulla, thymus, thyroid, salivary glands, skin and pituitary gland, and display marked variability in differentiation, proliferative activity, molecular background and clinical behaviour (1, 2). Current classification frameworks recognise well-differentiated neuroendocrine tumours (NETs) and poorly differentiated neuroendocrine carcinomas (NECs) as biologically distinct entities; however, important clinicopathological challenges remain, particularly in highly proliferative neoplasms and in settings where morphology, grade and biological aggressiveness do not fully coincide (2, 3).

Although pathological classification and clinical management of NENs have advanced substantially, biomarkers that adequately reflect biologically relevant tumour states remain limited. Histological grade and the Ki-67 proliferation index are central to prognostic stratification, but they do not fully capture tumour complexity and may be influenced by sampling bias and intratumor heterogeneity (2, 3). Likewise, conventional neuroendocrine markers are indispensable for confirming lineage and differentiation, yet offer only limited information regarding invasive behaviour, pathway activation and other biological features that may affect outcome and therapeutic vulnerability (4). This limitation is particularly relevant in endocrine and neuroendocrine pathology, where tumours with shared neuroendocrine differentiation may follow very different clinical courses (1, 4).

**Figure 1:**
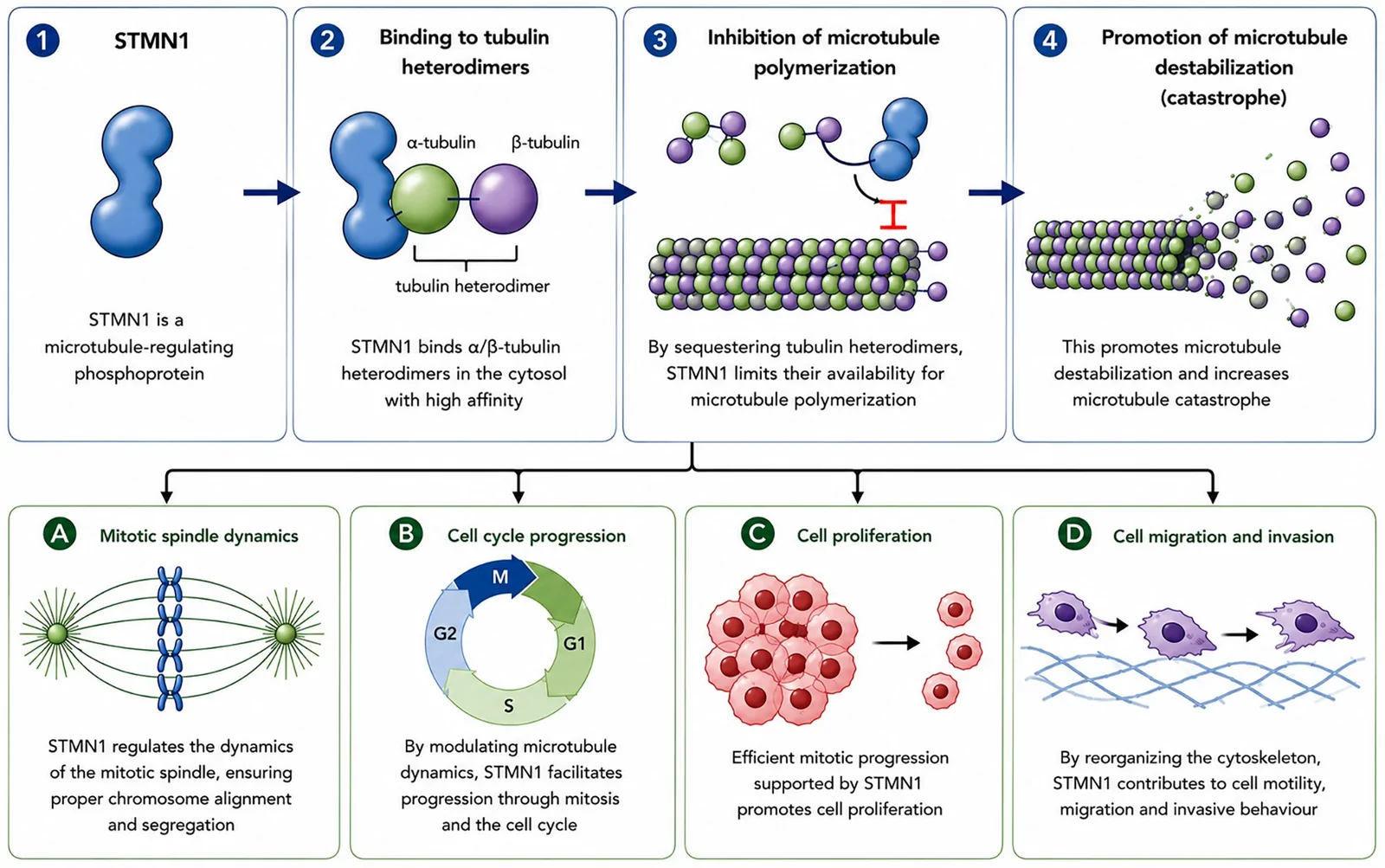
STMN1-mediated regulation of microtubule dynamics and cellular functions. STMN1 regulates microtubule dynamics through interaction with α/β-tubulin heterodimers, thereby limiting microtubule polymerization and promoting microtubule destabilization. Through its effects on cytoskeletal organization and mitotic spindle dynamics, STMN1 influences cellular processes including cell-cycle progression, proliferation, and migration. Upper panels (1–4) illustrate the molecular mechanism underlying these functions: (1) STMN1 is a microtubule-regulating phosphoprotein; (2) STMN1 binds α/β-tubulin heterodimers in the cytosol with high affinity; (3) sequestration of tubulin heterodimers limits their availability for microtubule polymerization; and (4) this promotes microtubule destabilization (catastrophe). Lower panels **(A–D)** depict the major downstream biological consequences of STMN1 activity: **(A)** regulation of mitotic spindle dynamics, ensuring proper chromosome alignment and segregation; **(B)** facilitation of cell-cycle progression through modulation of microtubule dynamics; **(C)** promotion of cell proliferation through efficient mitotic progression; and **(D)** enhancement of cell migration and invasion via cytoskeletal reorganization.

Stathmin 1 (STMN1), also known as oncoprotein 18, is a conserved cytosolic phosphoprotein involved in the regulation of microtubule dynamics by binding tubulin and promoting microtubule destabilisation and catastrophe (5, 6). Through these functions, STMN1 contributes to spindle organisation, mitotic progression and cytoskeleton-dependent cellular behaviour, processes that are broadly relevant to tumour proliferation, invasion and dissemination (5, 6). A schematic representation of the mechanism of action of STMN1 and its biological consequences is shown in **Figure 1**.

In several non-neuroendocrine malignancies, dysregulated STMN1 expression has been associated with adverse clinicopathological features and poorer clinical outcomes, suggesting that it may serve as a readout of biologically aggressive tumour states rather than simply reflecting lineage-specific differentiation (7–9).

These observations make STMN1 a plausible candidate for investigation in NENs. Its biological role is potentially relevant to neoplasms in which proliferative drive, cytoskeletal remodelling and aggressive clinical behaviour are of central importance, while currently available biomarkers remain only partially informative. However, the significance of STMN1 across neuroendocrine neoplasia remains uncertain. Existing studies have examined STMN1 in different endocrine and neuroendocrine contexts, including pulmonary, GEP, adrenal, cutaneous and pituitary neoplasms, but the available evidence is scattered across tumour sites, methodological platforms and clinical endpoints.

A focused synthesis is therefore warranted to examine whether STMN1 has meaningful biological and clinicopathological relevance in NENs, and to define the contexts in which it may be most informative. Rather than presuming a uniform role across neuroendocrine neoplasia, such an approach may clarify whether STMN1 represents an occasional finding in individual datasets or a more consistent signal deserving further pathological and translational investigation.

## 2 Aim

The aim of this narrative review is to map the available evidence on STMN1 in NENs across anatomical sites, to appraise whether reported associations with aggressive clinicopathological features are descriptive, independently validated, or functionally supported, and to assess whether current preclinical data provide a sufficient rationale for further site-specific translational investigation. To our knowledge, this represents the first narrative review specifically addressing the role of STMN1 in NENs.

## 3 Materials and methods

A literature search was conducted in PubMed, Web of Science, and Scopus from database inception to 25 January 2026. The search strategy combined terms related to STMN1, including “stathmin”, “stathmin 1”, “stathmin-1”, “oncoprotein 18”, and “Op18”, with terms related to neuroendocrine neoplasia across different anatomical sites. These included pulmonary neuroendocrine neoplasms (small-cell lung carcinoma, large-cell neuroendocrine carcinoma, typical carcinoid, and atypical carcinoid), gastroenteropancreatic neuroendocrine neoplasms, medullary thyroid carcinoma, pheochromocytoma and paraganglioma, pituitary neuroendocrine tumours, and Merkel cell carcinoma.

Original research articles published in English were considered eligible for inclusion. Reviews, editorials, letters, conference abstracts, and non-English language publications were excluded. Study selection was based on title and abstract screening, followed by full-text assessment of potentially relevant articles. Reference lists of selected studies were also screened to identify additional relevant publications.

Neuroendocrine prostate cancer and prostate adenocarcinoma with neuroendocrine differentiation were not included in the primary synthesis because the review scope was restricted to neuroendocrine neoplasms arising within the anatomical settings traditionally addressed by endocrine and neuroendocrine tumour classifications. These prostate entities are classified within the WHO Classification of Urinary and Male Genital Tumours (10) rather than within the endocrine/neuroendocrine NEN framework and are therefore acknowledged as biologically related but distinct contexts.

Given the narrative design of this review, no formal risk-of-bias assessment was undertaken, and no quantitative synthesis was performed. The findings are presented descriptively and are organised according to tumour site and study focus.

To facilitate interpretation of the heterogeneous literature, findings were considered according to whether they provided descriptive expression data, clinicopathological associations, functional preclinical observations, or direct mechanistic insights. This distinction is used narratively throughout the site-specific synthesis and revisited in the Discussion.

## 4 Evidence synthesis by anatomical site

The literature search ultimately yielded 12 original studies that met the inclusion criteria and were incorporated into the final narrative synthesis. The findings are presented below by anatomical site and integrated study focus.

### 4.1 Pulmonary neuroendocrine neoplasms

The pulmonary evidence base is the most extensive currently available and spans discovery proteomics, diagnostic validation and preclinical targeting, although not all components carry the same evidentiary weight, as summarised in **Table 1**.

**Table 1:** STMN1 in pulmonary neuroendocrine neoplasms.

Study	Experimental module	Methods	Biological material (n)	Principal finding	Study-specific context
Tanca, 2011 (11)	Proteomic discovery	GeLC-MS/MS; bioinformatics; IHC	6 FFPE lung neuroendocrine tumours (3 SCLC; 3 TC)	STMN1 enriched in SCLC versus TC; strong cytoplasmic staining confined to SCLC	Discovery proteomics, network placement and tissue confirmation in the same cohort
Yousefi, 2012 (12)	Secretome profiling	2DE; LC-MS/MS	Conditioned media from the QU-DB and Mehr-80 LCNEC cell lines	STMN1 identified in the conditioned-media secretome of LCNEC models; indirect relevance for pulmonary NENs	Supports extracellular recovery of STMN1 in LC models
Ocak, 2014 (13)	Membrane-associated proteomics and tissue validation	DIGE; LC-MS/MS (Orbitrap); Western blot; TMA; Cox regression	9 lung-derived cell lines; 136 lung tumours	STMN1 among the most overexpressed membrane-associated proteins in SCLC; increased serine-16 phosphorylation; higher expression in SCLC than NSCLC; not independently prognostic in multivariable survival analysis	PTM state associated to microtubule polymerization
Uribarri, 2014 (14)	Fluid biomarker development	2D-PAGE; MALDI-TOF/TOF MS/MS; xMAP	188 BAL samples (discovery); 252 BAL samples (validation)	STMN1 increased in SCLC BAL; STMN1 alone: Se 90%, Sp 52%, AUC 0.76; STMN1+GSTP1 panel: Se 90%, Sp 57%, AUC 0.80	Adjunctive fluid-based discrimination of SCLC versus NSCLC
Shimizu, 2019 (15)	Large-cohort diagnostic validation	IHC; qRT-PCR	552 thoracic neoplasms; 107 tumours for qRT-PCR	HGNETs (LCNEC and SCLC) showed uniformly high STMN1; qRT-PCR also discriminated HGNET from NSCLC	Robust diagnostic stratification in resections and biopsies
Yoshikawa, 2026 (16)	Genomic correlation and therapeutic targeting	RNA sequencing; copy-number analysis; IHC; targeted PIP repression; xenograft	213 LC cell lines; 94 mixed SCLC/NSCLC cases; SCLC cell lines and xenografts	STMN1 expression linked to copy-number alterations and suppressed by Chb-STMN1 PIP *in vitro* and *in vivo*, supporting preclinical proof-of-concept	Preclinical feasibility of STMN1 repression in SCLC models; further validation in primary systems and toxicological assessment are required before any clinical translation

2DE, two-dimensional electrophoresis; AUC, area under the curve; BAL, bronchoalveolar lavage; Chb, chlorambucil; DIGE, differential in-gel electrophoresis; FFPE, formalin-fixed paraffin-embedded; GeLC-MS/MS, gel electrophoresis-liquid chromatography tandem mass spectrometry; GSTP1, glutathione S-transferase pi 1; HGNET, high-grade neuroendocrine tumour; IHC, immunohistochemistry; LC, lung cancer; LC-MS/MS, liquid chromatography tandem mass spectrometry; LCNEC, large-cell neuroendocrine carcinoma; MALDI-TOF/TOF, matrix-assisted laser desorption/ionisation time-of-flight/time-of-flight; NSCLC, non-small-cell lung carcinoma; PIP, pyrrole-imidazole polyamide; PTM, post-translational modification; qRT-PCR, quantitative reverse-transcription polymerase chain reaction; SCLC, small-cell lung carcinoma; Se, sensitivity; Sp, specificity; TC, typical carcinoid; TMA, tissue microarray; xMAP, multi-analyte profiling.

Proteomic analysis of archival formalin-fixed paraffin-embedded (FFPE) lung NETs identified STMN1 as enriched in small-cell lung carcinoma (SCLC) relative to typical carcinoid (TC), thereby linking STMN1 to the high-grade pulmonary neuroendocrine phenotype from the earliest discovery phase (11). In the same study, bioinformatic interrogation placed STMN1 within cytoskeletal and high-grade molecular networks, and immunohistochemistry confirmed strong cytoplasmic expression in SCLC with absent staining in TC (11).

Secretome analysis of conditioned media from the large-cell lung carcinoma (LCNEC) cell lines QU-DB and Mehr-80 demonstrated extracellular detection of STMN1 (12). This study was conducted in LCNEC cell lines, suggesting that extracellular detection of STMN1 may be technically feasible and supporting its potential as a candidate marker for further validation in lung cancer body fluids.

Membrane-associated proteomic profiling across SCLC, non-small-cell lung carcinoma (NSCLC) and human bronchial epithelial control cell lines identified STMN1 as one of the most strongly overexpressed membrane-associated proteins in SCLC, with a mean differential ratio of 5.71 (13). Western blot analysis further showed increased phosphorylation at serine 16 in SCLC cell lines, indicating a post-translational state of STMN1 associated with microtubule dynamics in the high-grade neuroendocrine setting (13). Tissue microarray analysis confirmed significantly higher STMN1 expression in SCLC than in NSCLC, but multivariable survival modelling showed that STMN1 did not retain independent prognostic significance once histological subtype had been taken into account (13).

In bronchoalveolar lavage (BAL), discovery proteomics followed by Multi-Analyte Profiling (xMAP)-based validation (Luminex Corp., Austin, TX) showed that STMN1 was significantly increased in SCLC and could contribute to fluid-based diagnostic discrimination. However, optimal specificity was achieved when STMN1 was integrated into multi-marker panels rather than used as a standalone biomarker (14). Specifically, STMN1 alone distinguished SCLC from NSCLC with 90% sensitivity and 52% specificity, whereas the combined STMN1+GSTP1 model, that integrates the expression levels of both proteins to improve discrimination of tumor subtypes compared with either marker alone, achieved 90% sensitivity and 57% specificity (14).

Large-scale tissue validation confirmed the diagnostic usefulness of STMN1 in resection and biopsy material. In a cohort of 552 thoracic neoplasms, high STMN1 expression (modified Allred score 8) was consistently observed in high-grade neuroendocrine tumours (HGNETs; LCNEC and SCLC), whereas low-grade NETs and the great majority of NSCLCs showed low or intermediate staining; parallel quantitative reverse-transcription polymerase chain reaction (qRT-PCR) analyses also demonstrated markedly higher STMN1 transcript levels in HGNETs than in NSCLCs (15). Notably, recent large-scale evaluations employed automated immunohistochemical platforms and structured scoring systems.

More recently, genomic and functional work showed that STMN1 expression correlates with copy-number alterations and that chlorambucil-conjugated pyrrole-imidazole polyamide targeting of STMN1 can suppress tumour growth in SCLC models (16). These findings support the preclinical feasibility of STMN1-directed repression in SCLC, but they should be interpreted as proof-of-concept data rather than as evidence of near-term clinical applicability.

### 4.2 Pancreatic neuroendocrine neoplasms

In pancreatic neuroendocrine neoplasms, the available evidence is summarised in **Table 2**.

**Table 2:** STMN1 in pancreatic neuroendocrine neoplasms.

Study	Experimental module	Methods	Biological material (n)	Principal finding	Study-specific context
Schimmack, 2015 (17)	Copy-number analysis	qPCR	38 primary PanNENs; 5 metastases	No significant STMN1 copy-number gain	Overexpression is not copy-number driven
Schimmack, 2015 (17)	Transcript and protein expression	qRT-PCR; Western blot; IHC	Same cohort	Increased STMN1 mRNA and protein in tumour tissue versus normal pancreas	Concordant transcript and protein upregulation
Schimmack, 2015 (17)	Clinicopathological association	Correlation analysis	38 primary tumours	Higher STMN1 associated with larger tumour size and higher-grade disease, including greater expression in G3 than in G1 tumours	Links STMN1 to higher-grade and proliferative tumour behaviour, but without multivariable adjustment for major confounders such as Ki-67
Schimmack, 2015 (17)	Pathway modulation and functional silencing	PI3K inhibition; siRNA	BON-1 cells	Reduced serine-38 phosphorylation and reduced proliferation after PI3K inhibition; reduced proliferation after STMN1 knockdown	Supports a role for STMN1 downstream of PI3K-related proliferative signalling in BON-1 cells

G1, grade 1; G3, grade 3; IHC, immunohistochemistry; PanNENs, pancreatic neuroendocrine neoplasms; PI3K, phosphatidylinositol 3-kinase; qPCR, quantitative polymerase chain reaction; qRT-PCR, quantitative reverse-transcription polymerase chain reaction; siRNA, small interfering RNA.

Quantitative polymerase chain reaction (qPCR) analysis revealed no significant increase in STMN1 copy number in either primary tumours or metastatic lesions. In contrast, quantitative reverse-transcription polymerase chain reaction (qRT-PCR) demonstrated increased STMN1 mRNA expression in tumour tissue relative to normal pancreas, while western blotting and immunohistochemistry confirmed increased STMN1 protein expression. Clinicopathological analyses linked higher STMN1 expression to larger tumour size and to higher-grade disease, including significantly greater expression in grade 3 than in grade 1 tumours (17). However, these associations were not tested in multivariable models including established proliferative covariates such as Ki-67, so the evidence in primary PanNEN tissue remains predominantly associative. Interestingly, *in vitro* studies in BON cells demonstrated that STMN1 promotes cell proliferation, as its silencing reduces cell growth, while inhibition of the PI3K pathway decreases proliferation through STMN1 inactivation (dephosphorylation), supporting its functional role in tumor progression (17).

### 4.3 Pheochromocytoma and paraganglioma

The evidence regarding PCC and PGL is summarised in **Table 3**.

**Table 3:** STMN1 in pheochromocytoma and paraganglioma.

Study	Experimental module	Methods	Biological material (n)	Principal finding	Study-specific context
Sadow, 2008 (18)	Protein expression in primary tumours	TMA; IHC; Western blot	139 primary tumours; 5 samples for Western blot	Higher maximal STMN1 staining in malignant than in benign PCC; strongest Western blot band in malignant PCC	Supports association with malignant behaviour (i.e. PCC), but not stand-alone malignancy classification
Björklund, 2010 (19)	Transcript profiling	Microarray; qRT-PCR	11 samples	Sixteen-fold increase in STMN1 mRNA in metastatic tumours	Transcript-level enrichment in metastatic disease
Lin, 2011 (20)	Protein and transcript validation	IHC; Western blot; qRT-PCR	37 tumours	Higher STMN1 expression in malignant than in benign PCC (p = 0.01)	Independent confirmation of malignancy association
Szabó, 2012 (21)	Integrative systems analysis	Bioinformatic comparison of public datasets	Neuroblastoma versus PCC expression datasets	STMN1-centred signalling programme highlighted in the comparison; STMN1 higher in neuroblastoma than in PCC	Supports a STMN1-centred discriminatory programme in the neuroblastoma-vs-PCC comparison, with lower STMN1 expression in PCC than in neuroblastoma

IHC, immunohistochemistry; PCC, pheochromocytoma; PGL, paraganglioma; qRT-PCR, quantitative reverse-transcription polymerase chain reaction; TMA, tissue microarray.

Tissue microarray analysis of 139 primary tumours (28 malignant PCC, 48 benign PCC, 21 malignant PGLs, and 42 benign PGLs) showed significantly higher maximal staining intensity in malignant than in benign PCCs, while Western blotting demonstrated the strongest STMN1 band in the malignant PCC sample analysed; however, these findings do not support STMN1 as a stand-alone marker of malignancy (18).

Transcript-level studies supported the same biological direction. Microarray and qRT-PCR analyses demonstrated a 16-fold increase in STMN1 mRNA in metastatic tumours, and an independent series confirmed significantly higher STMN1 expression in malignant than in benign PCC/PGL (19, 20).

By contrast, integrative genomic comparison with neuroblastoma refined the interpretation of this signal by showing that the stathmin-centred pathway was prominent in the neuroblastoma-versus-PCC comparison, with STMN1 itself being expressed more strongly in neuroblastoma than in PCC. These data support STMN1 as part of a discriminatory signalling programme in the neuroblastoma/PCC contrast rather than as a simple PCC-specific overexpression marker (21).

### 4.4 Merkel cell carcinoma

The available evidence for MCC is summarised in **Table 4**.

**Table 4:** STMN1 in Merkel cell carcinoma.

Study	Experimental module	Methods	Biological material (n)	Principal finding	Study-specific context
Sadow, 2008 (18)	Baseline tumour expression	IHC	5 MCCs	All cases positive; 80% with strong (3+) staining	Defines high baseline STMN1 expression in MCC
Knight, 2015 (22)	Proteomic upregulation	SILAC proteomics; IHC	MCC cellular model; tissue validation	8.33-fold increase in STMN1 in the small-T-antigen model	Supports STMN1 upregulation in MCPyV-related MCC biology
Knight, 2015 (22)	Viral signalling mechanism	Co-immunoprecipitation; Western blot	MCPyV-positive MCC cell lines	Small T antigen associated with PP4C-mediated dephosphorylation of STMN1 at serine 16	Links viral signalling to STMN1 regulation
Knight, 2015 (22)	Functional silencing	siRNA	MCC cell lines	Reduced cell motility after STMN1 knockdown	Supports a preclinical role in migratory behaviour; therapeutic implications remain indirect

IHC, immunohistochemistry; MCC, Merkel cell carcinoma; MCPyV, Merkel cell polyomavirus; PP4C, protein phosphatase 4 catalytic subunit; SILAC, stable-isotope labelling with amino acids in cell culture; siRNA, small interfering RNA.

Immunohistochemical analysis showed positivity in all five MCCs examined, with strong staining in 80% of cases, thereby establishing a baseline pattern of high expression in this tumour type (18).

Mechanistic work in MCC cell systems reinforced this observation. Stable-isotope labelling with amino acids in cell culture (SILAC)-based proteomics demonstrated an 8.33-fold increase in STMN1 in a small-T-antigen-driven cellular model, and this finding was corroborated by tissue immunohistochemistry (22). In Merkel cell polyomavirus (MCPyV)-positive MCC cell lines, which are defined by the clonal integration of the MCC polyomavirus genome and the continued expression of viral small T and truncated large T antigens necessary for tumor cell proliferation, co-immunoprecipitation and Western blotting linked the viral small T antigen to protein phosphatase 4 catalytic subunit (PP4C), resulting in dephosphorylation of STMN1 at serine 16 (22). Functional STMN1 knockdown reduced cell motility, indicating that STMN1 contributes to the migratory phenotype of MCC (22).

### 4.5 Medullary thyroid carcinoma

The available evidence for MTC is summarised in **Table 5**.

**Table 5:** STMN1 in medullary thyroid carcinoma.

Study	Experimental module	Methods	Biological material (n)	Principal finding	Study-specific context
Sadow, 2008 (18)	Comparative thyroid tumour profiling	IHC	5 MTCs; 7 ATCs	STMN1 positive in 40% of MTCs, but in 100% of ATCs; strongest staining confined to ATC	Suggests limited expression in MTC

ATC, anaplastic thyroid carcinoma; IHC, immunohistochemistry; MTC, medullary thyroid carcinoma.

In thyroid neuroendocrine pathology, STMN1 expression appears limited in MTC but striking in anaplastic thyroid carcinoma (ATC). Immunohistochemical analysis showed STMN1 positivity in 2 of 5 MTCs (40%), and staining in this group was confined to weak or moderate intensity. By contrast, all 7 ATCs were strongly positive, with the highest-intensity staining observed exclusively in the anaplastic tumors. These findings suggest that while STMN1 is rarely expressed in MTC, it is a prominent feature of ATC, reflecting differences in tumor biology (18).

### 4.6 Pituitary neuroendocrine tumours

Only one study, summarised in **Table 6**, has investigated STMN1 expression in PitNETs. Importantly, the number of cases within each pituitary tumour subtype was very small in that study, including two null-cell adenomas, three gonadotroph adenomas, two prolactin adenomas, two growth hormone adenomas and three ACTH adenomas, in addition to two normal pituitaries. Therefore, the reported subtype-associated expression patterns should be interpreted as preliminary and not generalisable without validation in larger PitNET cohorts.

**Table 6:** STMN1 in pituitary neuroendocrine tumours.

Study	Experimental module	Methods	Biological material (n)	Principal finding	Study-specific context
Sadow, 2008 (18)	Subtype-restricted pituitary profiling	IHC	12 PitNETs; 2 normal pituitaries	No STMN1 in normal pituitary or prolactinomas; positivity in ACTH-secreting adenomas, gonadotroph tumours, GH adenomas and null-cell adenomas	Subtype-associated rather than universal pituitary expression

ACTH, adrenocorticotropic hormone; GH, growth hormone; IHC, immunohistochemistry; PitNET, pituitary neuroendocrine tumour.

Sadow and colleagues found no STMN1 immunoreactivity in normal anterior pituitary tissue or in prolactinomas, whilst expression was present in ACTH-secreting adenomas, gonadotroph tumours, growth hormone-secreting adenomas and, most consistently, null-cell adenomas (18).

Taken together, these findings suggest that, in pituitary neuroendocrine tumours, STMN1 may represent a preliminary subtype-associated signal in selected adenohypophyseal lineages rather than a general pituitary neuroendocrine marker (18).

## 5 Discussion

### 5.1 STMN1 across NENs: synthesis of the evidence and biological interpretation

Across anatomical sites, the available data show marked heterogeneity. Key findings are summarised in **Table 7**. Pulmonary NENs and Merkel cell carcinoma currently provide the clearest functional support for a biological role of STMN1, although the nature of this support differs between settings. Evidence in PanNENs remains primarily associative with limited functional validation, whereas observations in PCC/PGL, medullary thyroid carcinoma and pituitary neuroendocrine tumours are largely descriptive. This uneven distribution of data highlights both the potential relevance of STMN1 and the need for site-specific validation studies.

**Table 7:** Integrated overview across anatomical sites.

Anatomical district	Expression pattern	Quantitative evidence	Diagnostic utility	Therapeutic signal	Interpretive note
Lung	High in HGNETs, especially SCLC and LCNEC	MAS 8 in HGNETs; STMN1 alone Se 90% and Sp 52% in BAL; strong overexpression ratio 5.71 in membrane-associated proteomics	Diagnostic discriminator in tissue and BAL; therapeutic implications remain preclinical	STMN1-directed PIP showed proof-of-concept anti-tumour effects in SCLC xenografts	Most extensive evidence base, spanning discovery, diagnostics and preclinical targeting; not all signals carry the same evidentiary strength
Pancreas	Higher in larger and higher-grade PanNENs	Higher expression in grade 3 than grade 1 PanNENs; no relevant copy-number gain	Candidate marker of proliferative signalling and PI3K-pathway-related activity	Reduced proliferation after PI3K inhibition and STMN1 knockdown	Biologically coherent but based on a single study, with tissue-level associations not yet validated in multivariable analyses
Adrenal	Higher in malignant PCC/PGL than in benign counterparts	Malignant PCC mean maximal IHC 2.11; independent malignant series significant at p = 0.01	Potential adjunctive marker of malignant behaviour	No direct STMN1-targeted therapy reported	Potentially useful mainly as an adjunctive marker of malignant behaviour, not as a stand-alone classifier
Skin	Uniformly high in MCC	100% positive by IHC; 80% strongly positive; 8.33-fold protein increase in the viral model	Mechanistically linked to MCPyV-related signalling and tumour motility in preclinical models	Preclinical siRNA data support functional relevance	Strong expression with mechanistic viral linkage, but therapeutic implications remain preclinical
Thyroid	Limited in MTC, very high in ATC	MTC 40% positive; ATC 100% positive	Useful chiefly for contextual comparison with other endocrine tumours	No direct STMN1-targeted therapy reported	Primarily informative by contrast with ATC
Pituitary	Restricted to selected adenoma subtypes	Absent in normal pituitary and prolactinomas; present in ACTH, GH, gonadotroph and null-cell adenomas	Subtype-associated marker rather than pan-pituitary marker	No direct STMN1-targeted therapy reported	Subtype-selective rather than universal expression

ACTH, adrenocorticotropic hormone; ATC, anaplastic thyroid carcinoma; BAL, bronchoalveolar lavage; GH, growth hormone; HGNET, high-grade neuroendocrine tumour; IHC, immunohistochemistry; LCNEC, large-cell neuroendocrine carcinoma; MAS, modified Allred score; MCC, Merkel cell carcinoma; MTC, medullary thyroid carcinoma; PanNENs, pancreatic neuroendocrine neoplasms; PCC, pheochromocytoma; PGL, paraganglioma; PI3K, phosphatidylinositol 3-kinase; PIP, pyrrole-imidazole polyamide; SCLC, small-cell lung carcinoma; Se, sensitivity; siRNA, small interfering RNA; Sp, specificity.

The currently available literature suggests that STMN1 is a context-dependent biological signal that tends to emerge in NENs characterised by increased proliferative activity, higher grade or more aggressive clinical behaviour. At the same time, the current corpus should not be interpreted as a uniform body of evidence: some findings are descriptive, some are statistically associative, some derive from preclinical functional systems, and only a minority approach direct mechanistic inference.

A useful way to interpret the field is therefore to distinguish four levels of evidence: descriptive or associative evidence, based on immunohistochemistry or transcript expression without adjustment for major confounders; correlational evidence with multivariable support, when associations persist after statistical adjustment; functional preclinical evidence, when STMN1 modulation alters tumour behaviour *in vitro* or *in vivo*; and direct mechanistic evidence, when a defined molecular link to STMN1 regulation or action is demonstrated. This taxonomy is particularly important in NENs, where the literature is small and biologically heterogeneous.

Among all anatomical sites, the lung currently provides the most mature evidence base for STMN1. In pulmonary NENs, the signal has been reproduced across multiple experimental layers, including archival proteomics, fluid-based studies, tissue validation and preclinical targeting (11, 13–16). Even here, however, the evidence is not fully uniform: diagnostic support is stronger than prognostic support, and the data from Yousefi et al. should be regarded as indirect because they were generated in the conditioned-media secretome of LCNEC models (12).The pulmonary setting is particularly instructive because it illustrates how the biological meaning of STMN1 shifts according to platform and clinical application. In tissue-based studies, STMN1 is more useful as a discriminator of high-grade neuroendocrine phenotype than as a generic lineage marker; in bronchoalveolar lavage, it behaves as an adjunctive biomarker rather than a stand-alone test; and in SCLC models it currently represents a preclinical vulnerability signal rather than a validated therapeutic target.

Outside the lung, the evidence is more limited, but several site-specific patterns remain biologically coherent. In pancreatic neuroendocrine neoplasms, STMN1 represents one of the few extrapulmonary settings in which descriptive, clinicopathological and functional data coexist within the same study (17). Nonetheless, the tissue-level evidence remains predominantly associative, because independent prognostic value beyond grade and proliferative indices has not yet been demonstrated in multivariable analyses (17).In PCC and PGL, the available evidence supports a more restricted interpretation. Across protein- and transcript-level studies, STMN1 is consistently higher in malignant or metastatic disease (18–20), but the literature remains limited and does not yet establish whether STMN1 adds value beyond established pathological and clinical risk parameters.MCC represents a different, and in some respects stronger, extrapulmonary example. In this setting, STMN1 is not only highly expressed at baseline, but is also linked to a defined viral regulatory mechanism through MCPyV small T antigen and PP4C-dependent dephosphorylation (18, 22). This makes MCC one of the few settings in which STMN1 is supported by both functional and mechanistic evidence (22). Nevertheless, the translational implications remain preclinical, and current data support further biological investigation more than they support prioritising STMN1 as an immediate therapeutic target in MCC.Outside the scope of the present review, recent studies have reported increased STMN1 expression in neuroendocrine prostate cancer and prostate adenocarcinoma with neuroendocrine differentiation, including the study by Shi et al. (23). These entities are classified within the WHO Classification of Urinary and Male Genital Tumours rather than within the endocrine/neuroendocrine NEN framework (10). Although these entities are classified separately from the NEN settings considered here, such observations further support the broader association of STMN1 with proliferative activity, lineage plasticity and aggressive tumour phenotypes. Whether similar biological programmes operate across distinct neuroendocrine contexts remains to be established.Taken together, the cross-site evidence supports a broader conceptual interpretation: STMN1 may be more informative as a marker of tumour state than of tumour identity (11, 13, 17, 20, 22). Across different anatomical districts, increased expression tends to recur in aggressive, proliferative or poorly differentiated settings, but the reasons for this recurrence are not necessarily identical and should not be assumed to imply a shared causal role.

### 5.2 Therapeutic implications of STMN1: current evidence and future perspectives

Beyond its potential diagnostic and prognostic relevance, STMN1 may also represent a candidate translational target in selected neuroendocrine neoplasms, although current evidence remains almost entirely preclinical. Among NENs, the strongest therapeutic rationale derives from functional studies. In pulmonary NENs, repression of STMN1 expression reduced tumour growth in xenograft models (16). Similarly, studies in pancreatic neuroendocrine tumour systems have linked STMN1 expression to proliferative pathways involving PI3K/mTOR signalling (17), while in Merkel cell carcinoma STMN1 silencing reduced cellular motility and impaired migratory behaviour (22). Collectively, these observations suggest that STMN1 may contribute functionally to tumour progression rather than merely reflecting aggressive disease biology.

Although direct therapeutic evidence in NENs remains limited, studies performed in non-neuroendocrine malignancies have provided potentially informative insights. Elevated STMN1 expression has been associated with resistance to taxane-based chemotherapy in several tumour types (24, 25). In addition, kinase-dependent regulation of STMN1 activity, including phosphorylation events mediated through MAPK/ERK signalling, has been described in experimental models outside the NEN setting (26). Finally, context-dependent effects of STMN1 modulation on invasion and metastatic dissemination have been reported, highlighting the complexity of STMN1-directed therapeutic strategies (27).

Whether these observations can be translated to neuroendocrine neoplasms remains unknown. Nevertheless, the recurrent association of STMN1 with high-grade phenotypes, proliferative activity and aggressive biological behaviour across several NEN settings provides a rationale for future studies investigating STMN1 as a potential therapeutic vulnerability. Importantly, the evidence discussed above should be regarded as hypothesis-generating, since no STMN1-directed therapeutic strategy has yet undergone clinical validation in NENs.

A strength of the present synthesis is that it places pathological observations, biological findings and preclinical signals within a common interpretive framework rather than treating them as disconnected literature fragments. By explicitly distinguishing descriptive, associative, functional and mechanistic evidence, the review also clarifies where the field is relatively mature and where inference currently exceeds demonstration.

### 5.3 Knowledge gaps and future directions

Several limitations should be acknowledged. First, the evidence base is markedly unbalanced across tumour sites, with pulmonary NENs accounting for the largest and most methodologically diverse dataset. Second, many extrapulmonary observations derive from single studies or small cohorts. Third, very few studies formally tested whether STMN1 retains independent clinicopathological or prognostic significance after adjustment for key covariates such as grade, stage or Ki-67. Finally, most therapeutic implications remain preclinical and should not be overstated.

Future studies should prioritise: standardisation of STMN1 assessment, including reproducible immunohistochemistry scoring systems and externally validated cut-offs; multivariable analyses incorporating grade, stage and Ki-67 to test independent clinicopathological or prognostic value; subtype-focused functional studies in settings where the biological signal appears strongest; prospective validation of secretome- and BAL-based observations before any liquid-biomarker claims are advanced; and evaluation of whether STMN1 is more informative as a predictor of response to microtubule-directed therapies than as a direct therapeutic target. In addition, thymic NENs also warrant investigation. Despite their biological and classification similarities with lung NENs, STMN1 data in thymic tumours are limited to thymomas, with no studies addressing thymic carcinoids or neuroendocrine carcinomas.

## 6 Conclusions

STMN1 emerges as a recurrent, context-dependent molecular signal across several neuroendocrine neoplasm settings, more closely associated with proliferative activity, high-grade morphology and aggressive tumour behaviour than with neuroendocrine differentiation per se. Current evidence is strongest in pulmonary NENs and remains more limited or predominantly associative in most extrapulmonary settings. Therapeutic implications are still preclinical and should be considered hypothesis-generating. Further studies are needed to determine whether STMN1 provides independent prognostic information and whether STMN1-related biology may identify actionable vulnerabilities in selected aggressive NENs.
