# Supplementary material for: Stathmin 1 across neuroendocrine neoplasms: biological, pathological and translational insights
Source: Front Endocrinol (Lausanne). 2026 Aug 6;17:1846997. doi: 10.3389/fendo.2026.1846997 (PMC13491025; doi:10.3389/fendo.2026.1846997)
Supplement: Supplementary file 1 [file SupplementaryFile1.docx]

**Supplementary Table 1. STMN1 discovery proteomics and secretome profiling.**

| **Reference** | **Biospecimen** | **Platform** | **STMN1 finding** | **Quantitative data** | **Context** |
| --- | --- | --- | --- | --- | --- |
| Tanca, 2011 | FFPE lung neuroendocrine tumours | GeLC-MS/MS | STMN1 enriched in SCLC versus TC | Spectral counts 19 versus 1; RSC 2.577; p = 0.00295 | Archival tissue discovery proteomics |
| Yousefi, 2012 | Conditioned media from QU-DB and Mehr-80 cells | 2DE; LC-MS/MS | STMN1 identified among proteins detected in conditioned media from LCC cell lines; indirect relevance for pulmonary NENs | No validated STMN1-specific spot score reported in the main paper | Secretome profiling in large-cell lung carcinoma models, including one neuroendocrine variant (Mehr-80) |
| Ocak, 2014 | Membrane-associated lysates from lung-derived cell lines | DIGE; LC-MS/MS (Orbitrap) | STMN1 among the top overexpressed membrane-associated proteins in SCLC | Average ratio 5.71; p = 1.3 x 10^-12 | Cell-line discovery proteomics |
| Uribarri, 2014 | BAL discovery cohort | 2D-PAGE; MALDI-TOF/TOF MS/MS | STMN1 selected as a candidate for downstream validation | Advanced to xMAP validation in 252 BAL samples | Fluid-phase discovery proteomics |

Abbreviations: 2DE, two-dimensional electrophoresis; BAL, bronchoalveolar lavage; DIGE, differential in-gel electrophoresis; FFPE, formalin-fixed paraffin-embedded; GeLC-MS/MS, gel electrophoresis–liquid chromatography tandem mass spectrometry; LCC, large-cell lung carcinoma; LC-MS/MS, liquid chromatography tandem mass spectrometry; MALDI-TOF/TOF, matrix-assisted laser desorption/ionisation time-of-flight/time-of-flight; NENs, neuroendocrine neoplasms; RSC, log ratio of spectral counts; SCLC, small-cell lung carcinoma; TC, typical carcinoid; xMAP, multi-analyte profiling.

**Supplementary Table 2. Diagnostic performance metrics in fluid and tissue specimens.**

| **Reference** | **Analyte** | **Modality** | **Cohort** | **Cut-off** | **Se (%)** | **Sp (%)** | **AUC** | **p value** |
| --- | --- | --- | --- | --- | --- | --- | --- | --- |
| Uribarri, 2014 | STMN1 | xMAP | 252 BAL samples | Concentration threshold | 90 | 52 | 0.76 | 0.0005 |
| Uribarri, 2014 | STMN1+GSTP1 | xMAP model | 252 BAL samples | Modelled panel | 90 | **57** | 0.80 | N/A |
| Uribarri, 2014 | HPT | xMAP | 252 BAL samples | Concentration threshold | 95 | 58 | 0.85 | 5.0 x 10^-13 |
| Shimizu, 2019 | STMN1 | IHC | 453 resection specimens | MAS 8 | 100 | 98.8 | N/A | <0.001 |
| Shimizu, 2019 | STMN1 | IHC | 99 biopsy specimens | MAS 8 | 100 | 100 | N/A | <0.001 |
| Shimizu, 2019 | STMN1 | qRT-PCR | 107 tumours | 8.667 | 92.3 | 95.1 | 0.984 | <0.001 |
| Ocak, 2014 | STMN1 | TMA-IHC | 136 tumours | Median SCLC score 240 | N/A | N/A | N/A | <0.001 |

Abbreviations: AUC, area under the curve; BAL, bronchoalveolar lavage; GSTP1, glutathione S-transferase pi 1; HPT, haptoglobin; IHC, immunohistochemistry; MAS, modified Allred score; NSCLC, non-small-cell lung carcinoma; qRT-PCR, quantitative reverse-transcription polymerase chain reaction; SCLC, small-cell lung carcinoma; Se, sensitivity; Sp, specificity; TMA, tissue microarray; TMA-IHC, tissue microarray immunohistochemistry; xMAP, multi-analyte profiling. Note: for Ocak (2014), p < 0.001 refers to higher STMN1 expression in SCLC than in NSCLC by tissue microarray immunohistochemistry, and not to multivariable overall-survival analysis.

**Supplementary Table 3. Pharmacological and genomic targeting parameters.**

| **Reference** | **Agent/strategy** | **Target** | **Parameter** | **Value** |
| --- | --- | --- | --- | --- |
| Yoshikawa, 2026 | Chb-STMN1 PIP | STMN1 promoter/intron | Target coordinates | chr1:25907108-25907117 / chr1:25895061-25895070 |
| Schimmack, 2015 | BEZ235 | PI3K/mTOR | IC50 | 20-75 nM |
| Schimmack, 2015 | LY294002 | PI3K | IC50 | 10 uM |
| Knight, 2015 | siRNA | STMN1 mRNA | Knockdown efficiency | 80-90% |
| Knight, 2015 | Paclitaxel | beta-tubulin | Experimental concentration | 10 µM (*) |
| Knight, 2015 | Doxycycline | Inducible small T system | Induction concentration | 2 ug/mL |

Abbreviations**:** Chb, chlorambucil; IC50, half-maximal inhibitory concentration; mTOR, mechanistic target of rapamycin; PI3K, phosphatidylinositol 3-kinase; PIP, pyrrole-imidazole polyamide; siRNA, small interfering RNA. (*) *reported as 10 mM in the original paper, likely a typographical error for 10 µM*

**Supplementary Table 4. Clinical and pathological parameters in malignant PCC/PGL case series and comparative thyroid cohorts.**

| **Reference** | **Case/group** | **Age/sex / cohort summary** | **Size (cm)** | **Metastatic site** | **PASS** | **STMN1 readout** | **Outcome/summary** |
| --- | --- | --- | --- | --- | --- | --- | --- |
| Lin, 2011 | Malignant PCC #1 | 45/M | 5 | Regional/LN | 6 | 4 | Alive with disease |
| Lin, 2011 | Malignant PCC #2 | 38/F | 15 | LN/Lung | 6 | 4 | Alive with disease |
| Lin, 2011 | Malignant PCC #3 | 61/M | 10 | Regional/LN | 5 | 6 | Died with disease |
| Lin, 2011 | Malignant PCC #4 | 33/M | 5 | Regional/LN | 6 | 6 | Died with disease |
| Lin, 2011 | Malignant PCC #5 | 43/F | 5 | Regional/LN | 6 | 6 | Died with disease |
| Lin, 2011 | Malignant PGL #6 | 14/F | 5 | Regional/LN | 6 | 6 | Alive with disease |
| Lin, 2011 | Malignant PGL #7 | 21/M | 10 | Liver | 7 | 4 | Alive with disease |
| Lin, 2011 | Malignant PGL #8 | 21/M | 7 | Regional/LN | 5 | 4 | Alive with disease |
| Sadow, 2008 | Malignant PCC cohort (mean summary) | Mean age 44 years | N/A | Distant metastasis present in malignant cohort | N/A | Mean maximal STMN1 IHC 2.11 | Outcome not individually reported |
| Sadow, 2008 | MTC cohort (n = 5) | Group-level thyroid cohort | N/A | Primary tumours | N/A | 40% positive for STMN1 by IHC | Individual outcomes not reported |
| Sadow, 2008 | ATC cohort (n = 7) | Group-level thyroid cohort | N/A | Primary tumours | N/A | 100% positive for STMN1 by IHC | Individual outcomes not reported |

Abbreviations: ATC, anaplastic thyroid carcinoma; F, female; IHC, immunohistochemistry; LN, lymph node; M, male; MTC, medullary thyroid carcinoma; N/A, not applicable; PASS, pheochromocytoma of the adrenal gland scaled score; PCC, pheochromocytoma; PGL, paraganglioma.
